# Protective Effects of a Mixture of Propolis and *Petasites japonicus* Against UVB-Induced Photoaging Through Suppression of Oxidative Stress and MAPK/AP-1 Signaling

**DOI:** 10.3390/ijms27177702

**Published:** 2026-08-28

**Authors:** Jae Young Shin, Jun Hyeong Lim, Ji Hyeon Park, Byoung Ok Cho, Yong Kap Hur, Mi Yeung Kim, Hyun Kyung Lee, Seon Il Jang

**Affiliations:** 1 Institute of Health Science, Jeonju University, Jeonju 55069, Republic of Korea; sjy8976@naver.com (J.Y.S.); junhyeong0853@naver.com (J.H.L.); wlgusliza@naver.com (J.H.P.); enzyme21@naver.com (B.O.C.); 2 Research Institute, Uniquebiotech Co., Ltd., Iksan 54576, Republic of Korea; ubtc@uniquebiotech.co.kr (Y.K.H.); ubtc2@uniquebiotech.co.kr (M.Y.K.); 3 Department of Health Management, Jeonju University, Jeonju 55069, Republic of Korea; hklee25@jj.ac.kr

**Keywords:** *Petasites japonicus*, propolis, UVB-induced photoaging, oxidative stress, MAPK/AP-1 signaling

## Abstract

Ultraviolet B (UVB)-induced photoaging is associated with oxidative stress, inflammation, and extracellular matrix (ECM) degradation. This study investigated the protective effects of a mixture of propolis and *Petasites japonicus* (PPJM) against UVB-induced photoaging in SKH-1 hairless mice. HPLC analysis identified bakkenolide B, chrysin, and pinocembrin as the representative marker compounds of PPJM. Mice were orally administered PPJM (50, 100, or 200 mg/kg) daily for 6 weeks during repeated UVB irradiation (90 mJ/cm^2^, three times per week). PPJM significantly restored the glutathione, superoxide dismutase, and catalase levels that had been reduced by UVB exposure. PPJM also decreased dorsal skin tissue IL-1β and TNF-α levels while increasing hyaluronan levels. Histological analysis showed that PPJM attenuated UVB-induced epidermal thickening and collagen degradation. In addition, PPJM suppressed the expression of COX-2 and MMPs while increasing collagen type I and elastin expression. PPJM administration was also associated with reduced phosphorylation of p38 and JNK and with lower expression of c-Jun and FosB. These findings suggest that PPJM protects against UVB-induced photoaging in this preclinical model, in association with reduced oxidative stress and MAPK/AP-1-related ECM degradation.

## 1. Introduction

Skin photoaging is a complex biological process primarily caused by chronic exposure to ultraviolet (UV) radiation, particularly UVB [1]. Exposure to UVB radiation triggers the excessive generation of reactive oxygen species (ROS) within skin tissue, which in turn drives oxidative stress, inflammatory responses, and disruption of skin structure [2]. Persistent UVB exposure accelerates characteristic signs of photoaging, including wrinkle formation, epidermal thickening, reduced elasticity, and collagen degradation [3]. At the molecular level, UVB-derived oxidative stress engages mitogen-activated protein kinase (MAPK) signaling, most notably through c-Jun N-terminal kinase (JNK) and p38 MAPK, and this activation subsequently promotes the induction of activator protein-1 (AP-1) transcription factors such as c-Jun and FosB [4]. Activation of AP-1 promotes the expression of matrix metalloproteinases (MMPs), which contribute to extracellular matrix (ECM) degradation and collagen breakdown [5]. In addition, UVB exposure increases inflammatory mediators, including tumor necrosis factor-α (TNF-α), interleukin-1β (IL-1β), and cyclooxygenase-2 (COX-2), while reducing endogenous antioxidant defenses such as glutathione (GSH), superoxide dismutase (SOD), and catalase (CAT) [6]. Therefore, natural products with antioxidant and anti-inflammatory activities have attracted considerable attention as potential agents for preventing UVB-induced photoaging [7].

Recently, combinations of natural compounds have gained increasing interest because complementary interactions among bioactive constituents may improve pharmacological efficacy compared with single agents [8]. Such combinations may simultaneously target multiple pathological pathways involved in photoaging, including oxidative stress, inflammation, and ECM degradation [9].

Propolis is a natural resinous material produced by honeybees from plant resins, pollen, wax, and bee enzymes for hive protection [10]. It contains diverse bioactive compounds, including flavonoids and phenolic acids, and exhibits various pharmacological activities such as antioxidant, anti-inflammatory, antimicrobial, wound-healing, and immunomodulatory effects [11]. Previous studies have shown that propolis can suppress oxidative stress and inflammatory responses through modulation of multiple signaling pathways [12].

*Petasites japonicus*, commonly known as Japanese butterbur, is a perennial herb traditionally used in East Asia for the treatment of fever, pain, allergic disorders, asthma, and gastrointestinal diseases [13]. Recent studies have demonstrated that *P. japonicus* possesses antioxidant, anti-inflammatory, anti-allergic, neuroprotective, and anti-asthmatic activities, which are largely attributed to its phenolic and flavonoid constituents [14].

In our previous studies, a 1:1 (*w*/*w*) combination of propolis and *P. japonicus* exhibited greater antioxidant and anti-inflammatory activities than either extract alone, suggesting complementary biological effects between the two materials [15,16]. Specifically, this combination attenuated PM_2.5_-induced inflammatory responses in RAW264.7 macrophages [15] and NCI-H292 lung epithelial cells [16], and reduced airway inflammatory responses in mice with respiratory disease induced by co-exposure to PM_10_ and ovalbumin [17]. Furthermore, the propolis–*P. japonicus* mixture demonstrated neuroprotective efficacy by improving cognitive function and attenuating memory impairment in a scopolamine-induced mouse model [18]. Although these findings collectively support the broad therapeutic potential of this combination across respiratory and neurological systems, its protective effects against UVB-induced skin photoaging have not yet been investigated.

Accordingly, this study was designed to examine whether a combined preparation of propolis and *P. japonicus* (PPJM) confers protection against photoaging induced by UVB in SKH-1 hairless mice. To elucidate the underlying mechanisms, we evaluated oxidative stress markers, inflammatory cytokines, histopathological changes, ECM-related proteins, and MAPK/AP-1 signaling pathways in UVB-irradiated skin tissues.

## 2. Results

### 2.1. Quantification of Representative Marker Compounds in PPJM by HPLC Analysis

**Figure 1:**
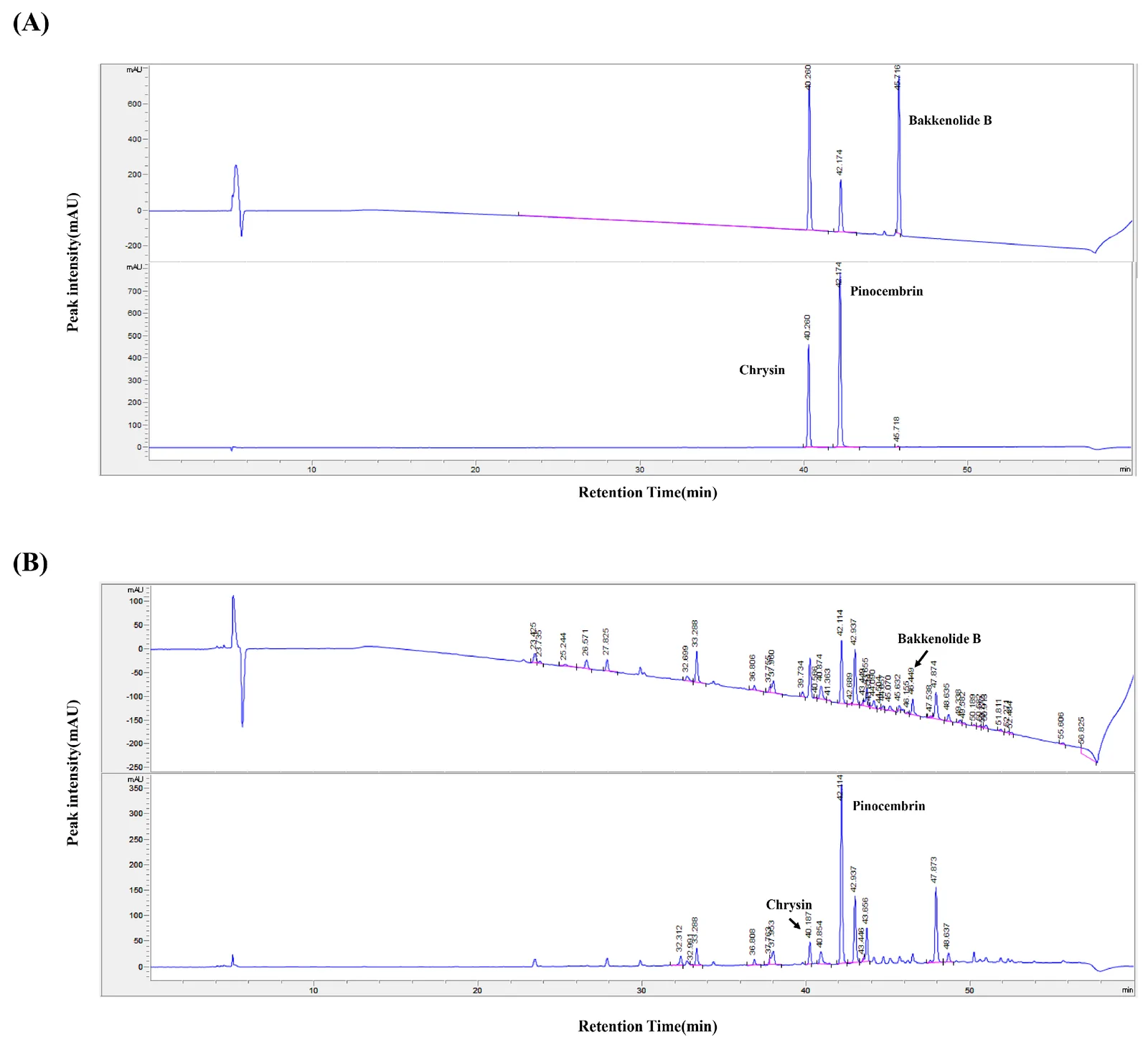
HPLC chromatograms of standard compounds and PPJM. (**A**) HPLC chromatograms of a standard mixture containing bakkenolide B (top, detected at 220 nm), chrysin, and pinocembrin (bottom, detected at 290 nm). (**B**) Representative HPLC chromatograms of PPJM obtained under the same analytical conditions. Arrows and labels indicate the peaks corresponding to bakkenolide B, chrysin, and pinocembrin, identified by comparison with the authentic standards shown in panel (**A**) (retention times 45.72, 40.26, and 42.17 min, respectively; Table 1). The remaining peaks were not identified in the present study. PPJM, mixture of propolis and *Petasites japonicus* extract.

To characterize the bioactive components of PPJM, HPLC-PDA analysis was conducted using standard reference compounds (Figure 1). Bakkenolide B, a characteristic sesquiterpene lactone from *Petasites japonicus*, was detected at a retention time of approximately 45.7 min (detected at 220 nm). Chrysin and pinocembrin, major flavonoids from propolis, were identified at retention times of approximately 40.3 min and 42.2 min, respectively (detected at 290 nm). These compounds were selected as representative marker compounds based on previous phytochemical studies of propolis and *P. japonicus*. Although additional chromatographic peaks were observed in Figure 1, these constituents were not structurally characterized in the present study. As shown in Table 1, the quantitative analysis revealed that PPJM contained 1246.54 ± 45.01 μg/g of bakkenolide B, 196.25 ± 2.79 μg/g of chrysin, and 637.70 ± 1.60 μg/g of pinocembrin. These findings confirm the presence of key bioactive constituents from both *P. japonicus* and propolis in the combined extract.

### 2.2. Protective Effects of PPJM on UVB-Induced Visual Skin Alterations

**Figure 2:**
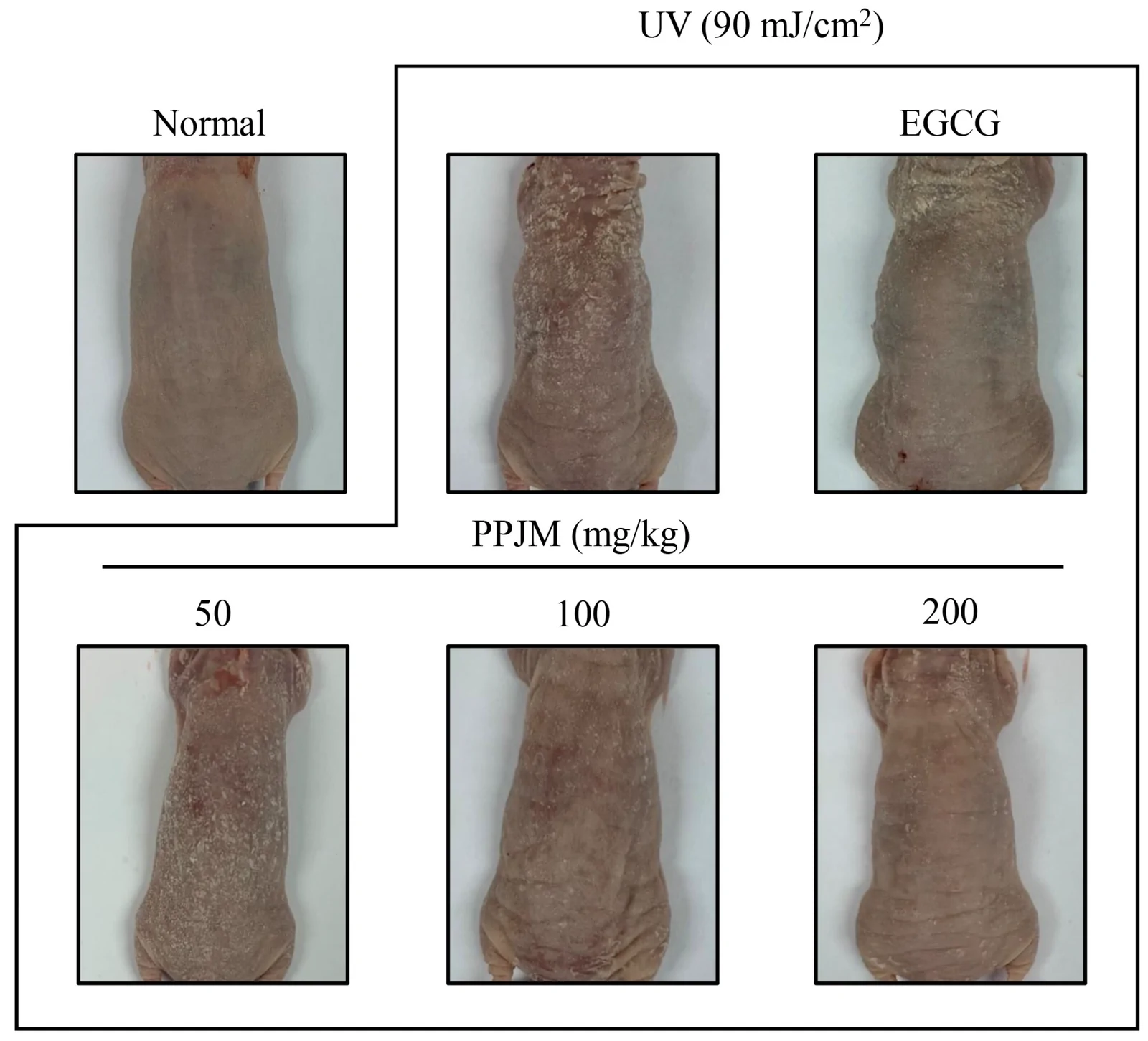
Effects of PPJM on macroscopic skin alterations in UVB-irradiated hairless mice. Representative photographs of the dorsal skin lesions showing changes in erythema, edema, deep wrinkles, and epidermal scaling.

To evaluate the macroscopic protective efficacy of PPJM against chronic UVB exposure, visual changes in the dorsal skin of SKH-1 hairless mice were monitored (Figure 2). Repeated UVB irradiation induced cutaneous alterations characterized by erythema, edema, wrinkle formation, and epidermal scaling in the UVB-alone group compared to the normal control group. In contrast, oral administration of PPJM at doses of 50, 100, and 200 mg/kg appeared to alleviate these photoaging symptoms, with greater effects observed at higher doses. Furthermore, the positive control group treated with EGCG (50 mg/kg) also showed an alleviation of UVB-induced skin damage. Representative photographs of the dorsal skin are shown in Figure 2. These images provide qualitative visualization of UVB-induced macroscopic changes, whereas quantitative evaluation of skin alterations was performed based on histomorphometric analyses, including epidermal thickness and dermal collagen density.

### 2.3. Histopathological Improvements and Preservation of Dermal Collagen by PPJM

**Figure 3:**
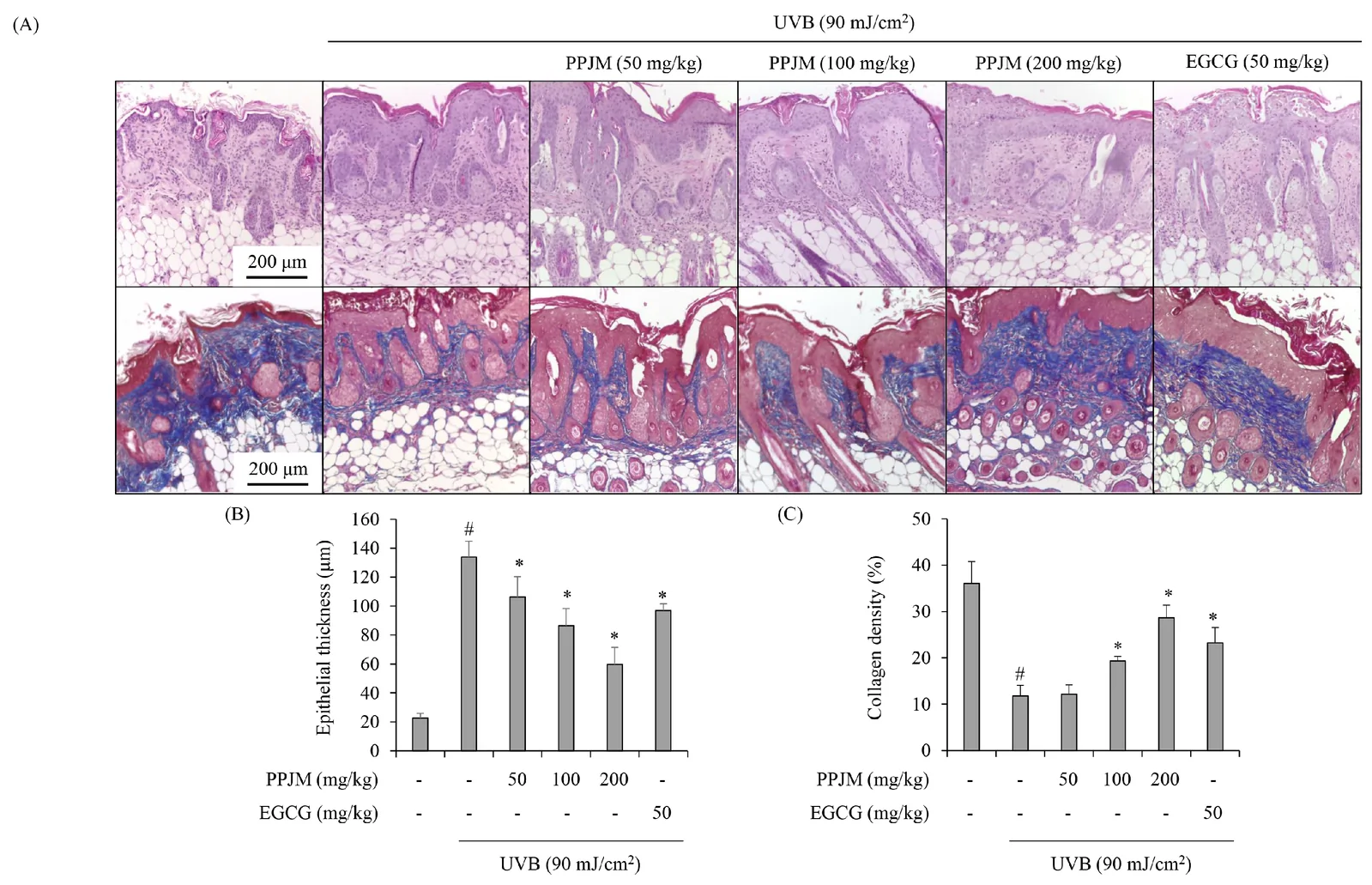
Effects of PPJM on histopathological changes and dermal collagen content in UVB-irradiated hairless mice. (**A**) Representative histopathological images of dorsal skin tissues stained with H&E and Masson’s trichrome. Quantitative analysis of (**B**) epidermal thickness and (**C**) dermal collagen density. Data are presented as mean ± SD (*n* = 6). # *p* < 0.05 vs. normal control group; * *p* < 0.05 vs. UVB-alone group.

Skin sections were subjected to H&E and Masson’s trichrome staining in order to assess microscopic and structural changes at the tissue level (Figure 3A). Repeated UVB irradiation led to an increase in epidermal thickness, a characteristic feature of epidermal hyperplasia. The epidermal thickness in the UVB-alone group was significantly higher than that in the normal control group (Figure 3B). However, oral administration of PPJM suppressed this UVB-induced epidermal thickening in a dose-dependent manner. The group treated with the highest dose of PPJM (200 mg/kg) exhibited a reduction in epidermal thickness, showing an approximately 55.4% decrease compared to the UVB-alone group. Masson’s trichrome staining was utilized to visualize the arrangement and density of collagen fibers in the dermal layer (Figure 3A). In the UVB-alone group, collagen density was significantly reduced, and the fibers appeared fragmented and sparsely distributed. In contrast, treatment with PPJM attenuated collagen degradation, showing a dose-dependent increase in collagen density (Figure 3C). The 200 mg/kg PPJM-treated group demonstrated a 143.8% increase in collagen density compared to the UVB-alone group. Additionally, the positive control group treated with EGCG (50 mg/kg) also exhibited a reduction in epidermal thickness and preservation of dermal collagen density. These findings were consistent with the histological observations.

### 2.4. Enhancement of Endogenous Antioxidant Enzyme Activities by PPJM

**Figure 4:**
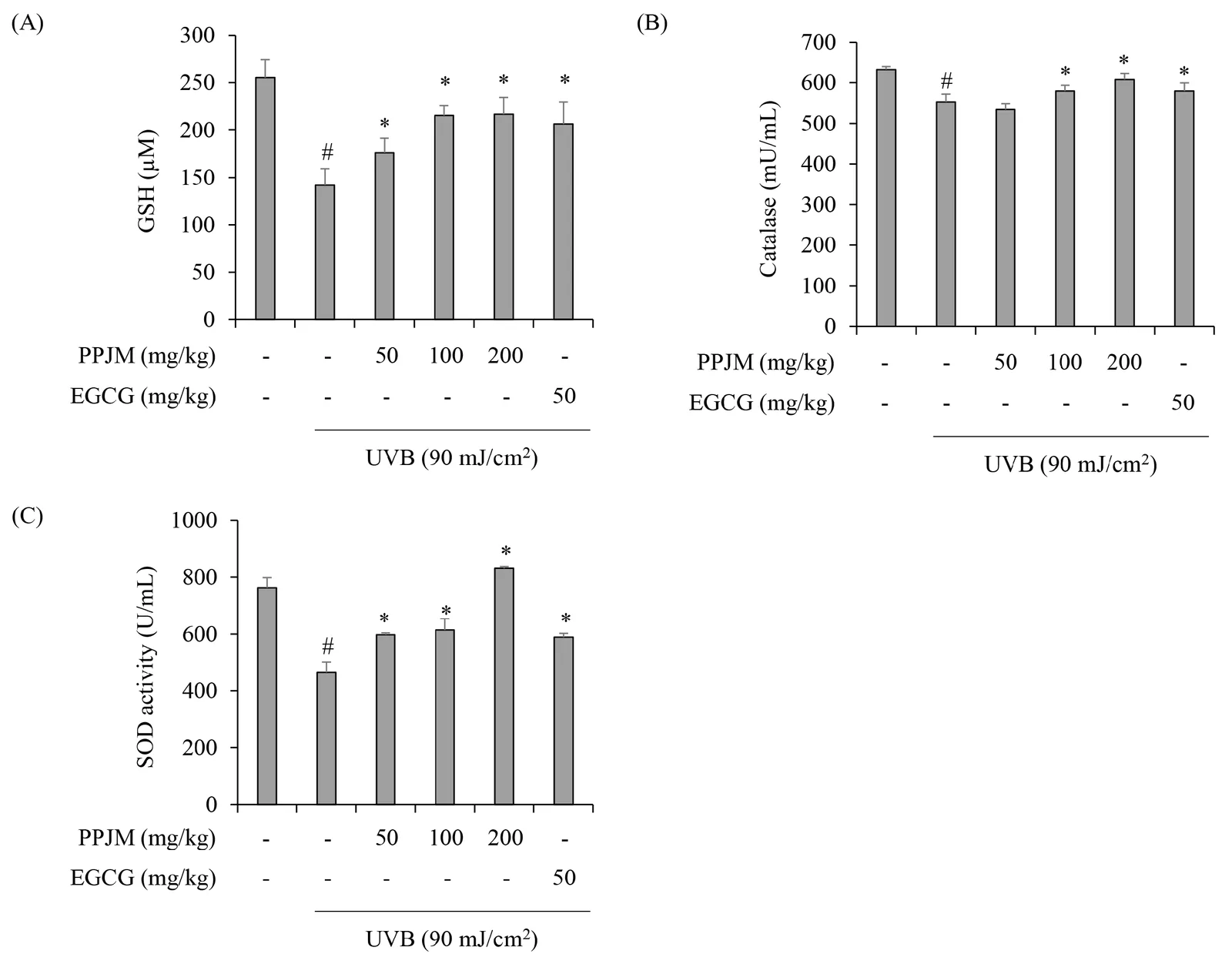
Effects of PPJM on endogenous antioxidant system in UVB-irradiated hairless mice. Levels or enzymatic activities of (**A**) GSH, (**B**) catalase, and (**C**) SOD in the skin tissues. Data are presented as mean ± SD (*n* = 6). # *p* < 0.05 vs. normal control group; * *p* < 0.05 vs. UVB-alone group.

Total GSH content along with CAT and SOD enzymatic activities were assessed in dorsal skin tissue in order to determine the extent to which PPJM suppresses UVB-induced oxidative stress (Figure 4). Chronic UVB irradiation impaired the endogenous antioxidant defense system, leading to a significant decrease in GSH levels (Figure 4A), CAT activity (Figure 4B), and SOD activity (Figure 4C) compared to the normal control group. In contrast, oral administration of PPJM progressively restored the depleted antioxidant defenses. The highest dose of PPJM (200 mg/kg) increased the levels or activities of GSH, CAT, and SOD by approximately 52.89%, 10.0%, and 78.0%, respectively, compared to the UVB-alone group. Furthermore, the EGCG-treated positive control group (50 mg/kg) also demonstrated a recovery in these endogenous antioxidant markers. These results demonstrated that PPJM restored the antioxidant defenses impaired by UVB exposure.

### 2.5. Regulation of Inflammatory Cytokines and Hyaluronan Levels by PPJM

**Figure 5:**
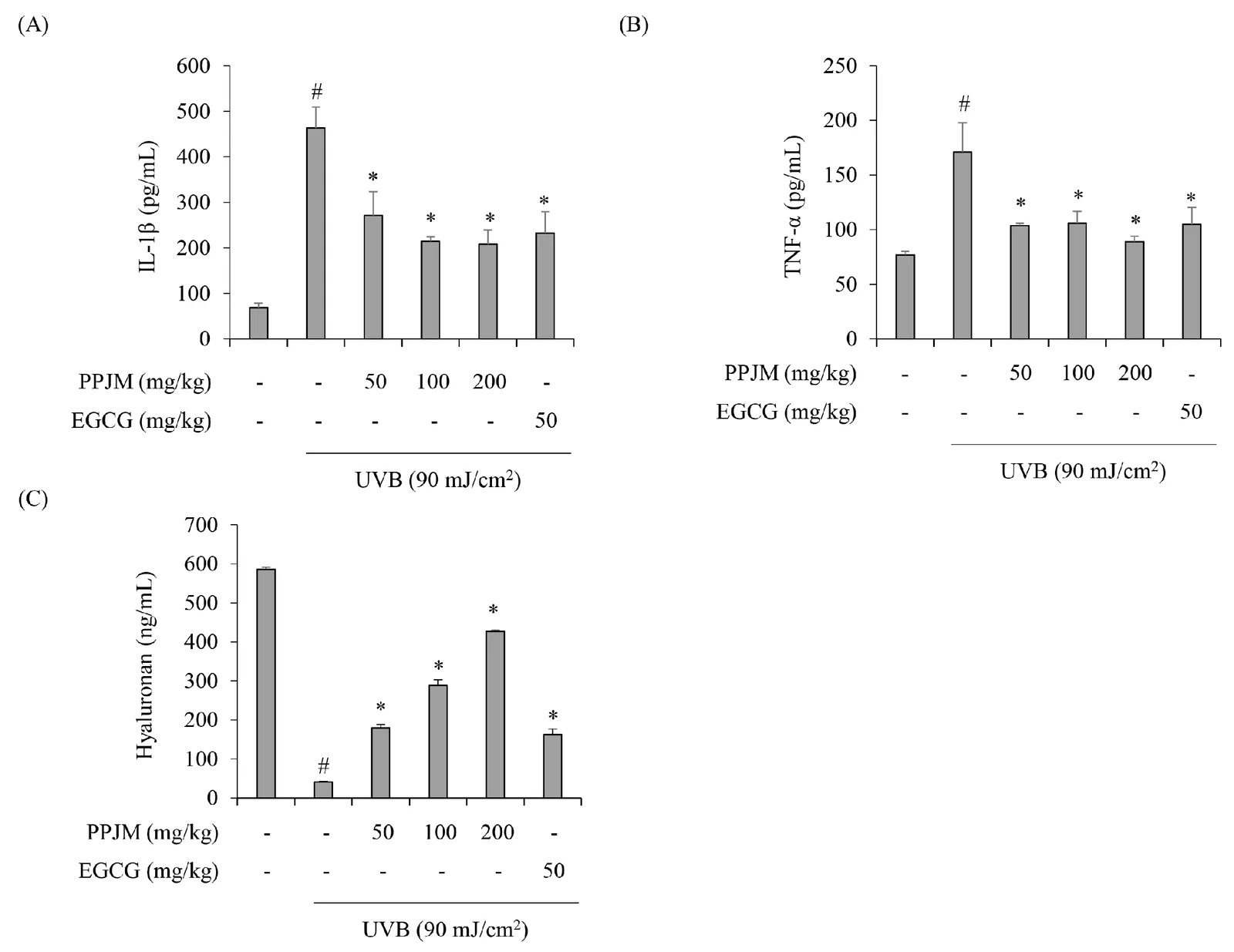
Effects of PPJM on pro-inflammatory cytokines and hyaluronan levels in UVB-irradiated hairless mice. Production levels of (**A**) IL-1β, (**B**) TNF-α, and (**C**) hyaluronan in the dorsal skin tissues measured by ELISA. Data are presented as mean ± SD (*n* = 6). # *p* < 0.05 vs. normal control group; * *p* < 0.05 vs. UVB-alone group.

ELISA was employed to quantify the IL-1β, TNF-α, and hyaluronan contents in dorsal skin tissue, thereby determining whether PPJM exerts anti-inflammatory and moisturizing properties (Figure 5). An inflammatory response was provoked by repeated UVB exposure, as reflected in the marked elevation of IL-1β (Figure 5A) and TNF-α levels (Figure 5B) relative to the normal control group. However, oral administration of PPJM decreased the production of these pro-inflammatory cytokines. In the 200 mg/kg PPJM-treated group, the levels of IL-1β and TNF-α were reduced by approximately 55% and 47%, respectively, compared with the UVB-alone group. Conversely, the level of hyaluronan decreased in the UVB-alone group (Figure 5C). PPJM treatment restored hyaluronan levels in a dose-dependent manner. The highest dose of PPJM (200 mg/kg) promoted hyaluronan recovery, restoring the level to approximately 90% of that observed in the normal control group. The positive control group treated with EGCG (50 mg/kg) also exhibited comparable inhibitory effects on inflammatory cytokine production and an increase in hyaluronan levels. Collectively, PPJM reduced IL-1β and TNF-α levels while restoring hyaluronan levels in UVB-irradiated skin.

### 2.6. Inhibition of ECM Degradation and MAPK/AP-1 Signaling Pathways by PPJM

**Figure 6:**
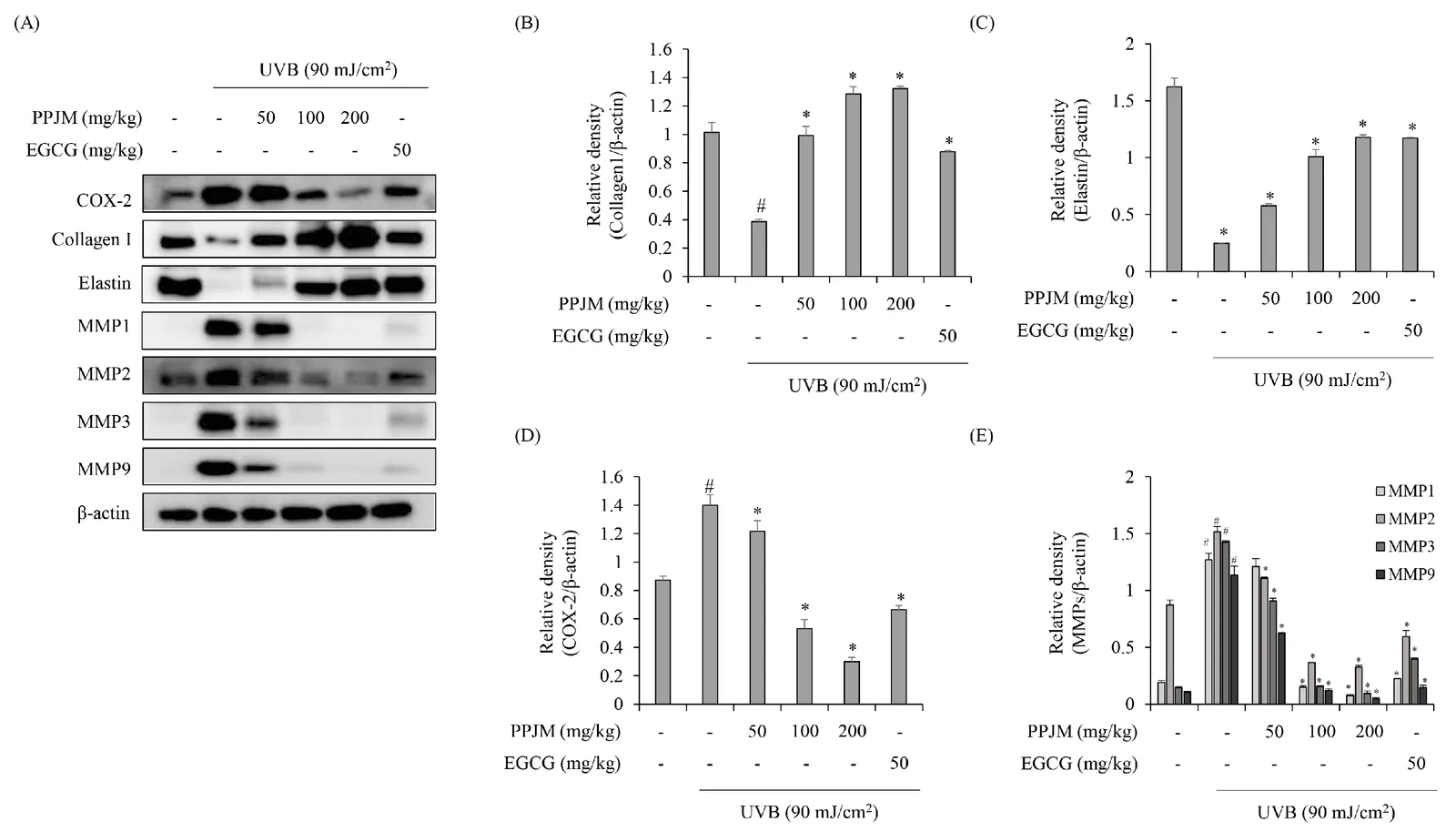
Effects of PPJM on ECM degradation markers and COX-2 expression in UVB-irradiated hairless mice. (**A**) Representative Western blot protein bands. Quantitative relative density analysis of (**B**) COX-2, (**C**) Collagen I, (**D**) Elastin, and (**E**) MMPs. Data are presented as mean ± SD (*n* = 6). # *p* < 0.05 vs. normal control group; * *p* < 0.05 vs. UVB-alone group.

**Figure 7:**
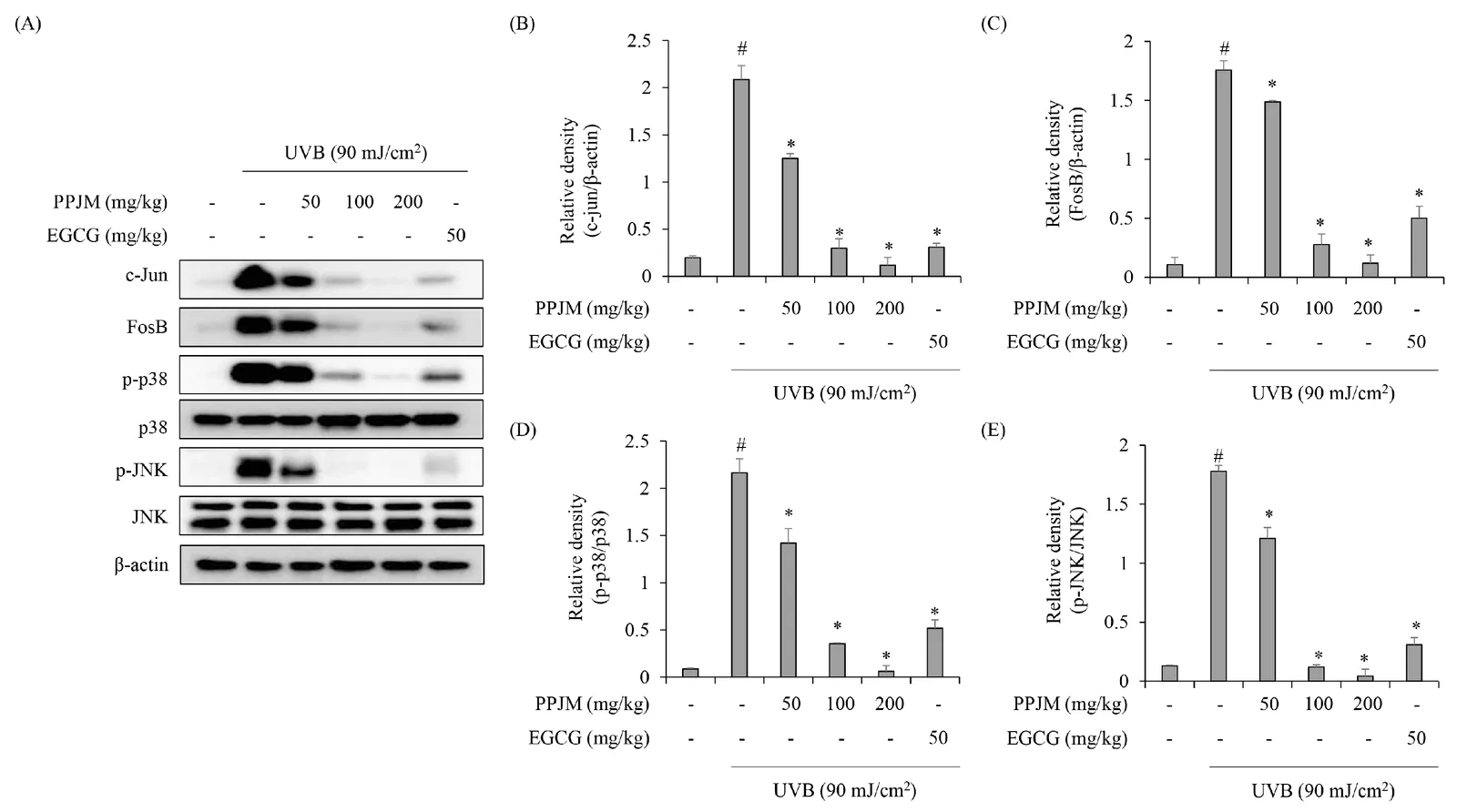
Effects of PPJM on MAPK/AP-1 signaling pathways in UVB-irradiated hairless mice. (**A**) Representative Western blot protein bands. Quantitative relative density analysis of the total protein levels of (**B**) c-Jun and (**C**) FosB, and the phosphorylation ratios of (**D**) p-p38/p38 and (**E**) p-JNK/JNK. Data are presented as mean ± SD (*n* = 6). # *p* < 0.05 vs. normal control group; * *p* < 0.05 vs. UVB-alone group.

To elucidate the molecular mechanisms underlying the protective effects of PPJM against UVB-induced skin aging, the expression levels of ECM regulatory proteins and the upstream MAPK/AP-1 signaling pathways were investigated using Western blot analysis (Figure 6 and Figure 7). UVB irradiation significantly decreased collagen I and elastin expression while increasing the expression of COX-2, MMP-1, MMP-2, MMP-3, and MMP-9 (Figure 6). However, oral administration of PPJM reversed these alterations in a dose-dependent manner. At the dose of 200 mg/kg, PPJM restored the expression of collagen I and elastin by approximately 71% and 79%, respectively, compared to the UVB-alone group. Concurrently, PPJM treatment decreased the UVB-induced upregulation of COX-2, MMP-1, MMP-2, MMP-3, and MMP-9 by up to 71%, 93%, 78%, 93%, and 95%, respectively. To examine changes in upstream signaling pathways, the activation of AP-1 transcription factors and MAPKs was evaluated (Figure 7). UVB irradiation increased the phosphorylation of p38 and JNK, which was accompanied by the upregulation of the AP-1 subunits, c-Jun and FosB. In contrast, PPJM administration decreased the phosphorylation of p38 and JNK, and lowered the expression of c-Jun and FosB in a dose-dependent manner. The maximum dose of PPJM (200 mg/kg) reduced the protein levels of c-Jun, FosB, p-p38, and p-JNK by 94%, 92%, 97%, and 97%, respectively, relative to the UVB-alone group. The positive control group treated with EGCG (50 mg/kg) also demonstrated a comparable inhibition of the MAPK/AP-1 pathway and recovery of ECM integrity. Collectively, PPJM decreased the expression of COX-2, MMPs, c-Jun, FosB, p-p38, and p-JNK while restoring collagen I and elastin expression.

## 3. Discussion

Chronic exposure to UVB radiation is a primary environmental factor responsible for skin photoaging, characterized by macroscopic alterations such as deep wrinkles, erythema, and epidermal hyperplasia, as well as microscopic damage to the dermal ECM [19]. A recent study demonstrated that a standardized hot water extract of *P. japonicus* leaves alone attenuated UVB-induced photoaging in hairless mice through the MAPK/AP-1 and TGF-β/Smad pathways [20]. The present study differs in that an ethanolic extract of *P. japonicus* was combined with propolis at a 1:1 ratio, and that the endogenous antioxidant enzyme system, inflammatory cytokines, hyaluronan, and multiple MMP subtypes were additionally evaluated. To the best of our knowledge, the anti-photoaging efficacy of a propolis—*P. japonicus* combination has not been reported previously. The protective effects of PPJM were supported by multiple findings, including histopathological improvement, enhancement of antioxidant defenses, suppression of inflammatory responses, and regulation of the MAPK/AP-1 signaling pathway.

The macroscopic and histopathological evaluations provided morphological evidence supporting the protective effects of PPJM. UVB exposure triggers epidermal hyperplasia as a defense mechanism against cellular damage, leading to an increase in epidermal thickness and the breakdown of dermal collagen fibers [21]. Our findings revealed that PPJM administration significantly suppressed epidermal thickening, achieving an approximately 55.4% reduction at 200 mg/kg, while concurrently enhancing dermal collagen density by 143.8%. This structural preservation was accompanied by an increase in hyaluronan levels, which were restored to approximately 90% of normal control levels in the PPJM-treated group. Hyaluronan is indispensable for maintaining skin hydration and viscoelasticity, and its robust recovery suggests that PPJM may effectively contribute to the maintenance of skin hydration and extracellular matrix integrity, which are typically disrupted by UV-induced damage [22].

At the molecular level, UV-induced skin damage is fundamentally initiated by the overproduction of ROS, which exhausts the endogenous antioxidant system and induces oxidative stress [23]. In our study, PPJM treatment was accompanied by a significant upregulation of vital endogenous antioxidant defenses, including GSH levels and the activities of catalase and SOD. This augmentation of the antioxidant defense system is crucial, as ROS act as secondary messengers that activate downstream pro-inflammatory and matrix-degrading pathways [24]. These changes were accompanied by reduced levels of IL-1β and TNF-α, changes consistent with attenuation of the chronic, low-grade cutaneous inflammation that accelerates tissue degradation during photoaging.

The regulation of MMPs via the MAPK/AP-1 signaling pathway represents one axis along which the protective effects of PPJM may be interpreted. UVB irradiation activates upstream MAPKs (specifically p38 and JNK), which in turn phosphorylate and activate the components of the AP-1 transcription factor complex, c-Jun and FosB [25,26]. Once activated, AP-1 translocates to the nucleus and drives the transcription of MMPs, including MMP-1 (collagenase), MMP-2 and -9 (gelatinases), and MMP-3 (stromelysin), leading to the comprehensive degradation of collagen I and elastin [27]. In this study, PPJM administration was associated with reduced phosphorylation of p38 and JNK and with lower levels of c-Jun (94% reduction) and FosB (92% reduction). These changes occurred concurrently with the downregulation of MMP-1, -2, -3, and -9, with reductions ranging from 78% to 95%, and with the preservation of collagen I and elastin expression. Because these signaling proteins and the phenotypic endpoints were measured concurrently at the end of the treatment period, and because no pathway-specific inhibitor or genetic loss-of-function approach was employed, these findings should be regarded as molecular changes associated with the protective effect of PPJM rather than as evidence that MAPK/AP-1 suppression is its causal mechanism.

The observed efficacy of PPJM may be explained by the complementary bioactivities of its constituents. In this study, quantitative HPLC analysis identified and quantified three representative marker compounds in PPJM: bakkenolide B (1246.54 μg/g), chrysin (196.25 μg/g), and pinocembrin (637.70 μg/g). Bakkenolide B, a characteristic sesquiterpene lactone from *P. japonicus*, is widely recognized for its potent anti-inflammatory properties that act through the inhibition of inflammatory signaling pathways [28]. Furthermore, chrysin, a representative flavonoid of propolis, has been reported to attenuate UV-induced ROS production, COX-2 expression, and JNK activation in human keratinocytes [29]; flavonoids of this class can also inhibit MMP activity in UVA-irradiated human dermal fibroblasts in proportion to their radical-scavenging capacity [30]. Pinocembrin, the most abundant propolis-derived flavonoid among the quantified propolis-related markers in PPJM, is one of the characteristic flavanones of poplar-type propolis and has long been recognized as a bioactive constituent of this material [11]. Beyond these flavonoids, propolis also contains caffeic acid phenethyl ester (CAPE), one of its most extensively characterized constituents. CAPE is a well-established inhibitor of NF-κB activation [31] and has been shown to suppress COX-2 expression and the production of TNF-α and IL-1β through the p38/ERK and NF-κB pathways [32]. In skin models, CAPE attenuated UV-induced MMP-1 expression in human dermal fibroblasts and human skin tissue, although this effect was attributed to epigenetic modulation of histone acetyltransferases rather than to inhibition of the canonical upstream kinases [33]. These reports indicate that propolis-derived phenolics can restrain both oxidative and inflammatory responses in skin. Consistent with these observations, previous studies from our group demonstrated that a 1:1 combination of propolis and *P. japonicus* extracts exhibited enhanced antioxidant and anti-inflammatory activities compared with the individual extracts [15,16]. These findings provide supporting evidence for the potential complementary benefits of combining these two botanical materials. However, because the present study did not include propolis-only or *P. japonicus*-only treatment groups, the relative contribution of each component and the nature of their interaction cannot be determined from the current data.

Accordingly, constituents derived from both components of PPJM may contribute through complementary and partially overlapping mechanisms. Compounds reported in *P. japonicus*, including sesquiterpene lactones such as bakkenolide B, and propolis-derived phenolics, such as pinocembrin, chrysin, and CAPE, have been associated with anti-inflammatory and antioxidant activities. Therefore, the relative contribution of each component cannot be determined from the present study, and the observed protective effects may reflect the combined actions of multiple constituents acting on oxidative and inflammatory pathways associated with photoaging. Because oxidative and inflammatory inputs can converge on p38/JNK-mediated AP-1 activation, modulation of these pathways by multiple constituents provides a plausible explanation for the concurrent restoration of GSH, CAT, and SOD levels/activities, the reduction in IL-1β and TNF-α levels, and the downregulation of MMPs observed in the present study. It should be noted that CAPE was not included among the marker compounds quantified in the present HPLC analysis, and its contribution is therefore inferred from previous reports rather than demonstrated directly in this study. Collectively, the quantitative confirmation of these representative marker compounds provides a phytochemical rationale for the photoprotective and anti-photoaging properties of PPJM. Although further studies, including comparisons with the individual extracts and clinical evaluation, are needed to confirm these mechanisms and to define the relative contribution of each component, the phytochemical composition of PPJM is consistent with the protective effects observed in this study.

Several limitations should be acknowledged. First, PPJM was only evaluated as a fixed 1:1 combination without propolis-only or *P. japonicus*-only comparator groups; therefore, the contribution of each component and their potential interaction could not be determined. Second, a PPJM-treated non-UVB group was not included, and the effects of PPJM on normal skin remain to be clarified. Third, all outcomes were assessed at a single experimental endpoint (end of week 6), and intermediate time-point analyses were not performed. Therefore, the temporal sequence and kinetics of the observed changes could not be determined. Accordingly, the observed changes in MAPK/AP-1 signaling should be interpreted as associated molecular changes rather than evidence of a causal mechanism. Fourth, the sample size was determined based on previous studies using similar UVB-induced photoaging models rather than an a priori power calculation. In addition, formal assessments of normality and homogeneity of variance were not performed, and effect sizes and confidence intervals were not estimated. Finally, systemic safety evaluation was limited to body weight monitoring and daily clinical observation, and comprehensive toxicological assessments were not performed. In addition, although residual L-arginine was present in the propolis preparation as a solubilizing agent, an arginine-only control group was not included; therefore, a minor contribution of arginine cannot be completely excluded. Further mechanistic, toxicological, and clinical studies are warranted.

## 4. Materials and Methods

### 4.1. Reagents

The reagents and antibodies used in this study were sourced from the following commercial suppliers. RIPA buffer (#89901), Halt™ Protease and Phosphatase Inhibitor Cocktail (#78440), collagen I antibody (PA1-26204), MMP-1 antibody (PA5-27210), and HRP-conjugated secondary antibodies (#31460 and #31430) were purchased from Thermo Fisher Scientific (Waltham, MA, USA). Commercial ELISA kits for IL-1β, TNF-α, and hyaluronan were obtained from R&D Systems (Minneapolis, MN, USA). The β-actin antibody (#4970) was supplied by Cell Signaling Technology (Danvers, MA, USA). Antibodies targeting elastin (sc-58756), MMP-2 (sc-13594), MMP-3 (sc-21732), MMP-9 (sc-393859), c-Jun (sc-74543), FosB (sc-8013), phospho-JNK (sc-6254), JNK (sc-7345), phospho-p38 (sc-7973), and p38 (sc-81621) were obtained from Santa Cruz Biotechnology (Santa Cruz, CA, USA). The COX-2 antibody (160126), together with GSH (703002) and SOD (706002) assay kits, were acquired from Cayman Chemical (Ann Arbor, MI, USA). Histological staining reagents, including an H&E staining kit and a Trichrome Stain Kit (ab150686), were purchased from Abcam (Cambridge, UK). A CAT assay kit (BO-CAT-400) and WestGlow™ FEMTO reagent (BWF0200) were provided by Biomax (Guri, Republic of Korea).

### 4.2. Preparation of P. Japonicus Leaf Extract and Propolis Extract

Leaves of *P. japonicus*, which were botanically authenticated, were collected during July 2025 at Cheonjam Mountain, situated in Jeonju, Republic of Korea. A voucher specimen (No. 2025-07-01) was subsequently deposited with the Department of Healthcare & Science, Jeonju University. The collected material was washed five times using distilled water and then air-dried in a shaded, well-ventilated location. A 100 g portion of this dried plant material was extracted with 2 L of 70% (*v*/*v*) ethanol for 48 h at room temperature. The resulting extract solution was filtered through a 0.5 μm membrane (ADVANTEC) and subsequently concentrated at 45 °C using a rotary evaporator (EYELA N-1100; Tokyo Rikakikai Co., Ltd., Tokyo, Japan), yielding 11.9 g of dried extract, corresponding to an extraction yield of 11.9%. The propolis extract was provided by Uniquebiotech Co., Ltd. (Iksan, Republic of Korea) and manufactured in accordance with Korean Patent No. 10-1403511, which describes the preparation of an alcohol-free, water-soluble propolis extract. Briefly, raw propolis was processed to remove wax and impurities, extracted with ethanol, and subsequently concentrated under reduced pressure. The resulting propolis concentrate was solubilized with polysorbate emulsifiers and L-arginine in purified water and dried into powder. L-arginine was used as a food-grade solubilizing agent to improve the water dispersibility of propolis resin constituents. PPJM was prepared by mixing the propolis extract and *P. japonicus* extract at a weight ratio of 1:1 (*w*/*w*), as previously described [15,16]. Based on the manufacturing input ratios specified in Korean Patent No. 10-1403511, the theoretical maximum L-arginine content of PPJM was estimated to be approximately 1.8% (*w*/*w*). Because the residual L-arginine content was not determined analytically, this value represents an upper-bound estimate derived from the manufacturing input ratios rather than a measured concentration.

### 4.3. High-Performance Liquid Chromatography (HPLC) Analysis

For chromatographic analysis, PPJM was prepared at a concentration of 10 mg/mL in methanol and passed through a 0.45 μm membrane syringe filter before injection. Quantitative analysis of the representative marker compounds (bakkenolide B, chrysin, and pinocembrin) in PPJM was performed using a Waters e2695 Alliance HPLC System (Waters Corp., Milford, MA, USA) equipped with a Waters 2998 photodiode array (PDA) detector. Separation of the target compounds was performed using an XBridge C18 column (4.6 mm × 250 mm, 5 μm; Waters Corp., Milford, MA, USA). The mobile phase consisted of 1% (*v*/*v*) acetic acid in HPLC-grade water (Mobile Phase A) and 1% (*v*/*v*) acetic acid in acetonitrile (Mobile Phase B). The gradient elution program was set as follows: 0–13 min, 10% B; 13–20 min, 10–25% B; 20–24 min, 25–30% B; 24–28 min, 30–35% B; 28–32 min, 35–45% B; 32–35 min, 45% B; 35–40 min, 45–50% B; 40–43 min, 50–55% B; 43–47 min, 55–60% B; 47–50 min, 60% B; and 50–55 min, 10% B. The flow rate and sample injection volume were fixed at 0.8 mL/min and 10 μL, respectively, and the column temperature was maintained at 35 °C. UV detection was monitored simultaneously at two wavelengths: 220 nm for bakkenolide B and 290 nm for chrysin and pinocembrin. Marker compounds were identified by comparing retention times with reference standards, and concentrations were calculated using linear calibration curves (R^2^ > 0.999). All analyses were performed in triplicate.

### 4.4. Animals and Experimental Design

**Figure 8:**
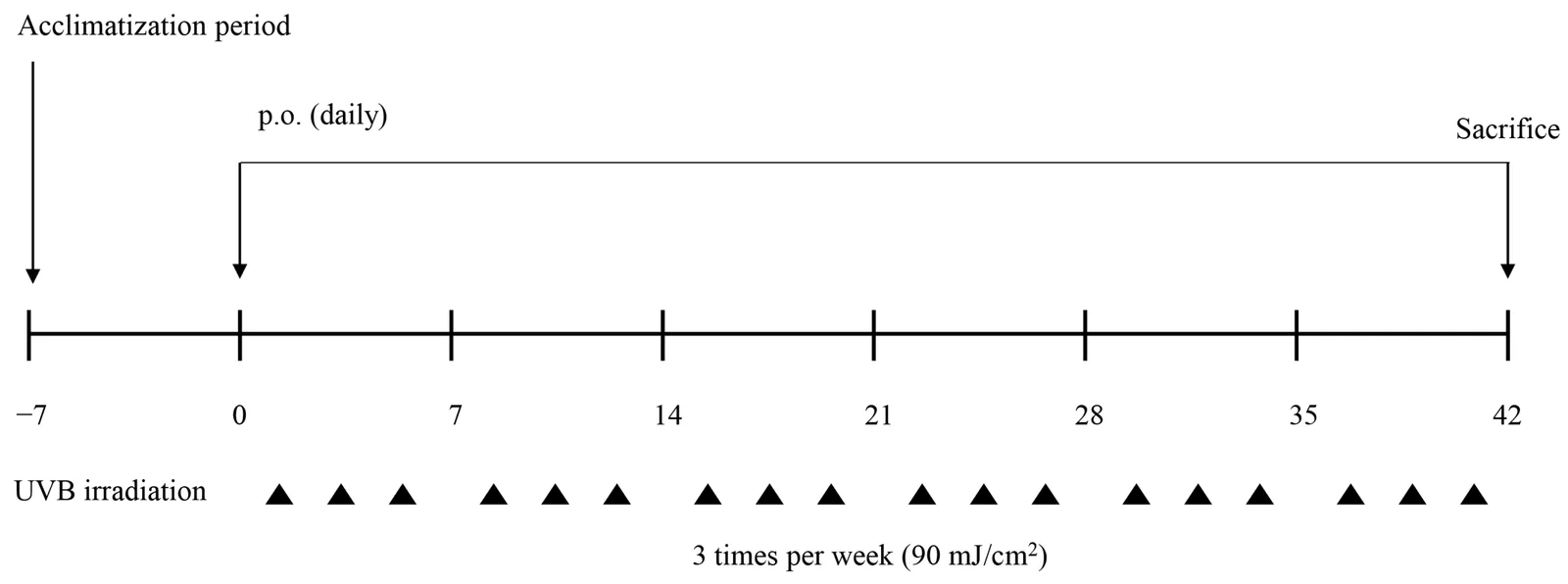
Experimental schematic outline and treatment schedule for the UVB-induced photoaging mouse model. Mice received oral administration (p.o.) of PPJM or vehicle once daily throughout the 6-week experimental period. UVB irradiation was performed three times per week (Monday, Wednesday, and Friday) for 6 weeks. Triangles indicate the days on which UVB irradiation was applied.

Approval for the present study was granted by the Institutional Animal Care and Use Committee (IACUC) of Jeonju University (Approval No. jjIACUC-20250902-2025-0902-A1). Male SKH-1 hairless mice, four weeks of age and specific pathogen-free, were obtained from Orient Bio Inc. (Seongnam, Republic of Korea). Prior to experimentation, animals underwent a 1-week acclimation period under controlled housing conditions (temperature, 22 ± 2 °C; humidity, 50 ± 10%; 12-h light/dark cycle), with unrestricted access to standard chow and water. Daily monitoring of animal health status was carried out throughout the study. Criteria for humane endpoints included body weight loss of 20% or more, reduced appetite persisting beyond two consecutive days, dyspnea, tachycardia, self-mutilation, jaundice, persistent diarrhea or vomiting, or diminished responsiveness to external stimuli; none of the animals met these criteria, and no unscheduled mortality or euthanasia occurred prior to the planned endpoint. After the 1-week acclimation period, the body weights of all animals were measured. Mice were stratified according to body weight and randomly assigned to experimental groups using a manual randomization procedure (drawing of lots). Because the investigator responsible for randomization and group allocation also administered the treatments, formal allocation concealment was not implemented, and blinding during treatment administration was likewise not achieved. The group size (*n* = 6 per group) was determined based on sample sizes commonly used in previous UVB-induced photoaging studies using SKH-1 hairless mice and in accordance with the 3R principle of animal reduction, as approved by the Institutional Animal Care and Use Committee. A formal a priori sample-size calculation was not performed. Photoaging was induced through UVB irradiation delivered at 90 mJ/cm^2^, administered three times per week over a 6-week period. Throughout this 6-week irradiation period, PPJM was administered orally on a daily basis at doses of 50, 100, or 200 mg/kg body weight. As a positive control, epigallocatechin gallate (EGCG, 50 mg/kg) was likewise administered daily via the same route. A schematic summary of the experimental design and timeline is provided in Figure 8. Following the completion of the 6-week experimental period, animals were euthanized humanely using a graduated CO_2_ inhalation system. Dorsal skin tissue was then promptly excised following euthanasia; the collected samples were either fixed in 10% neutral buffered formalin for subsequent histological examination or snap-frozen in liquid nitrogen and stored at −80 °C pending biochemical analysis. Histological analyses were performed by an investigator blinded to the group allocation. Western blot densitometric quantification was performed by the same investigator who was aware of the group allocation, and blinding was not applied at this step.

### 4.5. Measurement of Antioxidant Enzymes

To assess endogenous antioxidant defense systems, the activities of SOD and CAT, as well as the levels of GSH, were determined in the dorsal skin tissues. Briefly, the skin tissues were homogenized in an ice-cold lysis buffer, and the supernatants were collected by centrifugation at 12,000× *g* for 15 min at 4 °C to obtain total protein extracts. The total protein concentration of each sample was quantified using a BCA protein assay kit to normalize enzyme activities. The levels of GSH and the activities of SOD and CAT were measured using their respective commercial assay kits strictly according to the manufacturers’ instructions. All samples were analyzed in duplicate, and the absorbance was measured using a microplate reader.

### 4.6. Measurement of Inflammatory Cytokines and Hyaluronan

The levels of IL-1β and TNF-α and hyaluronan in the dorsal skin tissues were quantified using commercial ELISA kits according to the manufacturer’s instructions. Briefly, the total protein extracts obtained from the skin tissue homogenates—as described in the antioxidant enzyme assay section—were utilized for the analysis. The total protein concentration of each supernatant was predetermined using the BCA protein assay to ensure accurate normalization. No deviations from the manufacturers’ recommended protocols were made throughout the assay. Prior to analysis, samples were thawed on ice and each was tested in duplicate. A microplate reader was used to record absorbance at 450 nm, from which cytokine and hyaluronan concentrations were derived using their corresponding standard curves and subsequently normalized against total protein levels.

### 4.7. Hematoxylin and Eosin (H&E) Staining

For histological evaluation and epidermal thickness measurement, fixed dorsal skin specimens underwent dehydration, clearing, and paraffin embedding. Sections with 5 μm thickness were obtained from the paraffin blocks by microtome sectioning. Deparaffinization was carried out in xylene, followed by rehydration using a graded ethanol series (100%, 95%, 80%, and 70%) down to distilled water. Sections were then subjected to staining with Mayer’s hematoxylin (2 min), a bluing step under running tap water, and counterstaining with eosin Y (2 min). After a further dehydration and clearing sequence involving graded ethanol and xylene, sections were coverslipped using a permanent mounting medium. Histological images were acquired using a light microscope. For each animal, a single paraffin block was prepared from the same region of the dorsal skin. Epidermal thickness was quantified by measuring the distance from the stratum granulosum to the dermo-epidermal junction at 5 to 10 randomly selected, non-overlapping sites within the analyzed sections using ImageJ software (version 1.53e; National Institutes of Health, Bethesda, MD, USA). The measurements obtained from these sites were averaged to generate a single value for each animal. The animal, rather than the individual measurement site, was used as the experimental unit throughout, and all statistical analyses were performed on these per-animal means (*n* = 6 mice per group). All measurements were performed by an investigator blinded to group allocation.

### 4.8. Masson’s Trichrome Staining

To evaluate collagen degradation and density in the dermal ECM, Masson’s trichrome staining was performed using a Trichrome Stain Kit (ab150686; Abcam, Cambridge, UK) according to the manufacturer’s instructions. Briefly, paraffin-embedded skin sections (5 μm thick) were deparaffinized and rehydrated through graded ethanol solutions. The sections were refixed in Bouin’s fluid at 56 °C to improve staining quality, followed by staining with Weigert’s iron hematoxylin, Biebrich scarlet-acid fuchsin, phosphomolybdic-phosphotungstic acid, and Aniline blue reagents supplied in the kit according to the manufacturer’s recommended staining conditions. After differentiation in 1% acetic acid, the sections were dehydrated, cleared, permanently mounted, and observed under a light microscope. The collagen-stained blue area in the dermis was quantified relative to the total dermal area using ImageJ software. For each animal, a single paraffin block was prepared from the same region of the dorsal skin, and 5 to 10 randomly selected, non-overlapping fields from the analyzed sections were quantified. The values obtained from these fields were averaged to yield a single value per animal. The animal, rather than the individual field, was used as the experimental unit throughout, and all statistical analyses were performed on these per-animal means (*n* = 6 mice per group). All measurements were performed by an investigator blinded to group allocation.

### 4.9. Western Blot Analysis

Total proteins were extracted from frozen mouse skin tissues using ice-cold RIPA buffer supplemented with protease and phosphatase inhibitor cocktails. Protein lysates were prepared from individual mice, and six independent biological samples were analyzed per group (*n* = 6 mice/group). The protein concentration was determined using a BCA protein assay kit. Equal amounts of protein lysates (30 μg per lane) were separated by 8–12% SDS-PAGE and transferred onto PVDF membranes. For each target protein, one sample from each experimental group was loaded on the same membrane to allow for direct comparison among groups, and the analysis was repeated until all biological samples were evaluated. All membranes were processed using the same antibody incubation conditions and detection procedures. The membranes were blocked with 5% skim milk in TBST at room temperature for 1 h and then incubated overnight at 4 °C with specific primary antibodies against COX-2, collagen I, elastin, MMP-1, MMP-2, MMP-3, MMP-9, c-Jun, FosB, p-p38, p38, p-JNK, JNK, and β-actin. The following day, the membranes were washed five times with TBST and incubated with HRP-conjugated secondary antibodies for 2 h at room temperature. After five additional washes with TBST, protein bands were visualized using the ALLIANCE LD4 imaging system (Uvitec, Cambridge, UK) with an ECL chemiluminescent reagent. Band densities were quantified using ImageJ software (National Institutes of Health). Total protein levels were normalized to β-actin, whereas phosphorylated protein levels were normalized to the corresponding total protein levels. Densitometric values obtained from each individual mouse were used for statistical analysis. Representative blot images are presented in Figure 6A and Figure 7A.

### 4.10. Statistical Analysis

All statistical analyses were performed using SPSS version 30.0 (IBM Corp., Armonk, NY, USA). Data are presented as mean ± standard deviation (SD), with each animal considered the experimental unit (*n* = 6 per group). Differences among groups were analysed using one-way ANOVA followed by Tukey’s HSD post hoc test. Tukey’s HSD was selected because it controls the family-wise error rate for pairwise comparisons within each endpoint. A *p*-value < 0.05 was considered statistically significant. Formal correction for multiple testing across different categories of biological endpoints was not applied.

## 5. Conclusions

Oral administration of PPJM attenuated UVB-induced skin photoaging in SKH-1 hairless mice. PPJM restored the endogenous antioxidant defenses that had been depleted by UVB irradiation, lowered IL-1β and TNF-α levels while increasing hyaluronan content in dorsal skin tissue, preserved epidermal thickness, dermal collagen, and elastin, and suppressed COX-2 and MMP expression. These protective effects were accompanied by reduced phosphorylation of p38 and JNK and by lower expression of c-Jun and FosB, indicating that they are associated with modulation of MAPK/AP-1-related signaling. Because the present study was conducted in a preclinical rodent model using a fixed 1:1 combination without single-extract comparator groups, the relative contribution of each component and the causal role of the MAPK/AP-1 pathway remain to be defined. Further mechanistic, toxicological, and clinical studies are therefore required before the anti-photoaging potential of PPJM in human skin can be established. PPJM represents a promising candidate ingredient for functional foods or nutraceuticals intended to protect the skin against UVB-induced photoaging.

## Figures and Tables

**Table 1:** HPLC analytical parameters and contents of representative marker compounds in PPJM.

Compound	Retention Time (min)	Linearity (R^2^)	Content (μg/g Extract)
bakkenolide B	45.72	0.9999	1246.54 ± 45.01
chrysin	40.26	0.9998	196.25 ± 2.79
pinocembrin	42.17	0.9999	637.70 ± 1.60

## Data Availability

The original contributions presented in this study are included in the article. Further inquiries can be directed to the corresponding author.

## Abbreviations

The following abbreviations are used in this manuscript:

AP-1	Activator protein-1
CAT	Catalase
COX-2	Cyclooxygenase-2
ECM	Extracellular matrix
EGCG	Epigallocatechin gallate
ELISA	Enzyme-linked immunosorbent assay
GSH	Glutathione
H&E	Hematoxylin and eosin
HRP	Horseradish peroxidase
IL-1β	Interleukin-1 beta
JNK	c-Jun N-terminal kinase
MAPK	Mitogen-activated protein kinase
MMP	Matrix metalloproteinase
PPJM	Mixture of propolis and *Petasites japonicus*
PVDF	Polyvinylidene difluoride
RIPA	Radioimmunoprecipitation assay
ROS	Reactive oxygen species
SDS-PAGE	Sodium dodecyl sulfate-polyacrylamide gel electrophoresis
SOD	Superoxide dismutase
TBST	Tris-buffered saline containing Tween 20
TNF-α	Tumor necrosis factor-alpha
UV	Ultraviolet
UVB	Ultraviolet B
